# Cerebral Blood Flow Measurements in Adults: A Review on the Effects of Dietary Factors and Exercise

**DOI:** 10.3390/nu10050530

**Published:** 2018-04-25

**Authors:** Peter J. Joris, Ronald P. Mensink, Tanja C. Adam, Thomas T. Liu

**Affiliations:** 1 Department of Nutrition and Movement Sciences, NUTRIM School of Nutrition and Translational Research in Metabolism, Maastricht University Medical Center, 6200 MD Maastricht, The Netherlands; r.mensink@maastrichtuniversity.nl (R.P.M.); t.adam@maastrichtuniversity.nl (T.C.A.); 2 Center for Functional Magnetic Resonance Imaging (MRI), University of California San Diego, La Jolla, CA 92093-0677, USA; ttliu@ucsd.edu

**Keywords:** arterial spin labeling, brain health, cerebral blood flow, cognitive function, diet, exercise, magnetic resonance imaging, vascular function

## Abstract

Improving cerebrovascular function may be a key mechanism whereby a healthy lifestyle, of which a healthy diet combined with increased physical activity levels is a cornerstone, protects against cognitive impairments. In this respect, effects on cerebral blood flow (CBF)—a sensitive physiological marker of cerebrovascular function—are of major interest. This review summarizes the impact of specific dietary determinants and physical exercise on CBF in adults and discusses the relation between these effects with potential changes in cognitive function. A limited number of randomized controlled trials have already demonstrated the beneficial effects of an acute intake of nitrate and polyphenols on CBF, but evidence for a relationship between these effects as well as improvements in cognitive functioning is limited. Moreover, long-term trans-resveratrol supplementation has been shown to increase CBF in populations at increased risk of accelerated cognitive decline. Long-term supplementation of *n-3* long-chain polyunsaturated fatty acids may also increase CBF, but related effects on cognitive performance have not yet been found. Significant decreases in cerebral perfusion were observed by commonly consumed amounts of caffeine, while alcohol intake was shown to increase CBF in a dose-dependent way. However, the long-term effects are not clear. Finally, long-term exercise training may be a promising approach to improve CBF, as increases in perfusion may contribute to the beneficial effects on cognitive functioning observed following increased physical activity levels.

## 1. Introduction

The World Health Organization has estimated that by the year 2050, the world population aged 60 years or older is expected to total two billion, up from 900 million in 2015 [1]. Human aging is associated with an increased risk for developing severe medical conditions, including cognitive decline, dementia, and cardiovascular disease (CVD). As a result, the medical, psychosocial and economic consequences directly related to aging are enormous, and effective intervention strategies are needed for the prevention or delayed progression of these age-related conditions. The American Heart Association has now concluded that a healthy lifestyle, of which a healthy diet combined with increased physical activity levels is a cornerstone, protects against cognitive impairments [2] and CVD outcomes [3]. Therefore, increasing attention has been directed towards dietary factors and types of physical exercise that may account for these positive health effects. However, the number of well-designed randomized controlled trials (RCTs) with hard clinical disease outcomes (e.g., the PREvención con DIeta MEDiterraneá (PREDIMED) trial [4]) is very limited, as they require the inclusion of thousands of study participants with very long follow-up periods. As an alternative approach, the effects on risk markers for disease can be investigated, such as vascular function markers. In fact, improving brain vascular function may be a key mechanism to a healthy lifestyle by preventing many of the age-related conditions [2,3].

Compared to the effects of dietary factors and physical exercise on peripheral vascular markers, which are associated with future cardiovascular events and also used to demonstrate CVD benefits [5], not much is known about their effects on the vasculature of the human brain. This is of particular interest, since impaired brain vascular function is a key pathological event that precedes the development of impaired cognitive function [6,7]. In this respect, the impact on cerebral blood flow (CBF)—a sensitive physiological marker of cerebrovascular function [8,9]—can be assessed. Normal human aging is associated with a progressive decline in cerebral perfusion, and changes in CBF are associated with changes in cognitive function and the risk of developing Alzheimer’s disease and other types of dementia [2].

**Figure 1:**
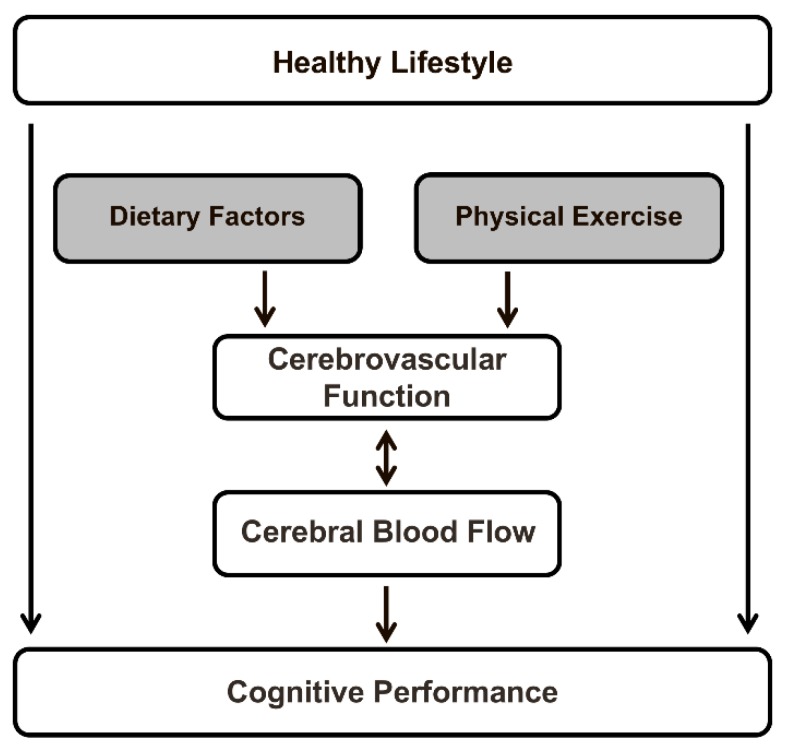
Schematic overview of the relationships described in the present review paper. The impact of both specific dietary factors (i.e., dietary nitrate, polyphenols, dietary fatty acids, caffeine, and alcohol) and physical exercise on cerebral blood flow (CBF)—a sensitive physiological marker of vascular function in the human brain—is summarized, and the relation between these effects in adult subjects with changes in cognitive performance is examined.

The aim of this review is to discuss the effects of dietary factors and exercise on CBF in adults. First, we will briefly describe the relation between CBF with cognitive function and the different techniques currently used to quantify global and regional cerebral perfusion. We will then discuss RCTs investigating the effects of different dietary factors on cerebral perfusion in adults and examine the relation between these effects and changes in cognitive performance (Figure 1). The focus will be on dietary nitrate, polyphenols, dietary fatty acids, caffeine, and alcohol, since the effects of other dietary factors on brain health in humans have hardly been studied [10]. Besides dietary determinants, we will also briefly review the effects of physical exercise on cerebral perfusion. Finally, possible future research directions in the field will be discussed.

## 2. Cerebral Blood Flow and Cognitive Function

The human brain requires a constant movement of blood through a network of cerebral arteries and veins to deliver oxygen, glucose, and other essential nutrients, but also to remove carbon dioxide, lactic acid, and other metabolic products. CBF in adults represents approximately 15% of the total cardiac output, while the brain accounts for only 2% of total body weight. Regional blood flow, which is tightly regulated to meet the metabolic demands of the brain, varies significantly between gray and white matter, and among different gray matter regions [11]. After adolescence, cerebral blood flow stays relatively stable for a long period, after which it steadily declines. In fact, in middle-aged and elderly adults, aging accounts for a decrease of approximately 0.45% to 0.50% in global CBF per year [12,13,14]. It has been shown that, likewise, perfusion through both cortical regions of the cerebral cortex decreases with age, especially in the frontal, temporal, and parietal lobes, and subcortical regions [14]. Aging is also a main risk factor for cognitive impairment and dementia. In elderly subjects, regional CBF in the superior temporal gyrus was positively associated with global cognitive performance [15]. Furthermore, lifestyle factors (e.g., dietary composition and physical exercise) increased global and regional CBF, and these lifestyle-induced changes in cerebral perfusion may improve cognitive functioning. These relationships are schematically depicted in Figure 1.

Differences in CBF between elderly subjects are related to vascular risk factors as well as risk factors for dementia [14]. Bangen and colleagues have observed that the presence of multiple vascular risk factors may add to the already diminished cerebral perfusion that results from aging [16]. It has also been shown that mean gray matter CBF was 15% lower in late middle-aged subjects (mean age 60 years) suffering from metabolic syndrome than age-matched healthy subjects, and was associated with lower cognitive function [17]. Moreover, in the elderly, reduced cerebral perfusion correlated with the volume of white matter hyperintensities [18] and cortical microbleeds [19], which are established risk factors for dementia. Neurovascular coupling is another critical component that affects CBF and consequently cognitive function. This phenomenon refers to the close temporal and regional relationship between (local) neural activity elicited, for example, by a cognitive task and subsequent changes detected in cerebral perfusion. In particular, aging impairs the mechanisms that match oxygen and nutrient delivery with the increased metabolic demands in active brain regions [20]. Many human intervention studies have therefore focused on middle-aged and elderly adults at increased vascular risk who are also known to be at increased risk of cognitive impairment and dementia [21], allowing for improvement by lifestyle-based intervention strategies.

## 3. Measurements of Cerebral Blood Flow

CBF is defined as the volume of arterial blood delivered to a unit mass of brain tissue per unit of time. The different imaging techniques to assess brain perfusion will be mentioned briefly, as they have already been critically reviewed [22]. The direct methods discussed below have been developed to measure the delivery of arterial blood to the capillary bed. The standard unit of measurement is milliliters (mL) of blood per 100 grams (g) of brain tissue per minute (min). A frequently observed value in human gray matter is about 60 mL/100 g/min, corresponding to the delivery of approximately 1 mL of blood to 100 g of brain tissue per second. Assuming an average brain tissue density of 1 g/mL, this means that approximately 1% of the total tissue volume is provided with freshly delivered blood each second [8]. In earlier studies, radioactive tracers were used to measure the absolute blood flow in the brain. However, CBF measurements using radiotracers require a specialized imaging unit, and time intervals between repeated measurements are also required to minimize overexposure to radiation. These delays significantly reduce the usefulness of radioactive CBF measurements in human intervention studies. Therefore, increasing attention has now been directed to recent developments in magnetic resonance imaging (MRI) that enable the non-invasive measurement of cerebral perfusion in human volunteers [8,9].

### 3.1. Direct Methods

**Figure 2:**
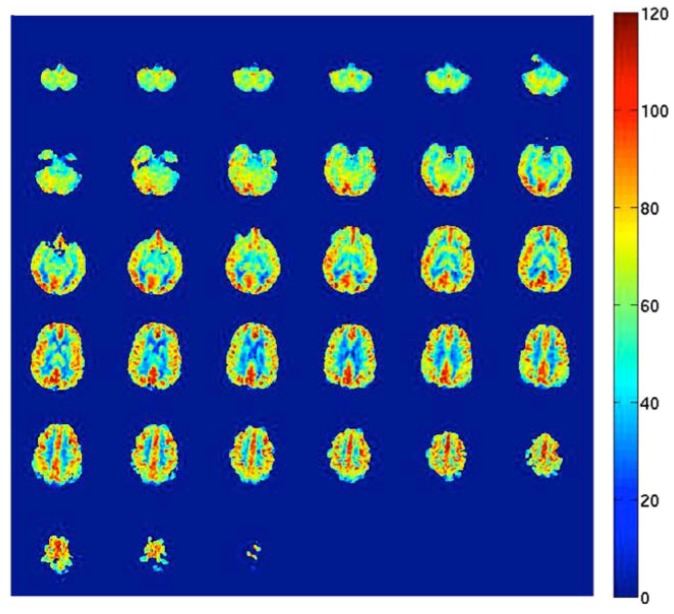
Arterial Spin Labeling (ASL) cerebral blood flow map in units of milliliters of blood per 100 grams of brain tissue per minute (mL/100 g tissue/min).

Direct methods for measuring CBF in human subjects include, but are not limited to, single-photon emission computed tomography (SPECT), positron emission tomography (PET), MRI with contrast agents, and arterial spin labeling (ASL) MRI. All these methods are based on the measurement of the amount of a tracer delivered to the human brain tissue by blood flow [8,9]. PET using injection of ^15^O-labeled water radiotracers is still considered the gold standard approach. Important limitations, however, include the need for an on-site cyclotron (the inherent radiation dose of the radiotracer) and the invasive nature and complexity of the measurement. As a promising alternative [23], ASL is a relatively new, non-invasive MRI method that uses magnetically labeled water molecules in arterial blood as a tracer. This method is currently available on MRI systems produced by the major manufacturers. The general principles have been described in detail before [8]. Briefly, this non-invasive measurement of CBF works by manipulating the magnetic resonance signal of inflowing blood in feeding arteries before it is delivered to the capillary bed of the different areas in the brain. Separate “label” and “control” images (with and without prior labeling of arterial blood) are acquired, and the resulting signal difference can be scaled to yield highly repeatable quantitative measures of CBF [8,9]. Figure 2 shows an example of an ASL CBF map. Recently, human studies that performed both PET and ASL MRI to measure brain perfusion were systematically reviewed. It was concluded that ASL is a promising method for accurate and reproducible CBF measurements, and comparative studies show that ASL is a validated method for non-invasive perfusion imaging in humans [23].

### 3.2. Indirect Methods

Indirect methods for the non-invasive assessment of CBF include transcranial Doppler ultrasound imaging (i.e., sonography), phase-contrast MRI, and near-infrared spectroscopy (NIRS). These techniques indirectly measure cerebral perfusion and cannot provide a direct measure of the blood that has been delivered to a brain region.

Using an ultrasound probe, it is possible to measure through thinner areas of the skull and record the blood flow velocity in the major cerebral arteries [24]. The most frequently used measurement site is the trans-temporal window, which is the thinnest part of the temporal bone, where the middle cerebral artery (MCA)—one of the three major paired arteries that supply blood to the brain—can be identified. The mean blood flow velocity is defined as the average velocity during one cardiac cycle. It is important to note that arterial blood flow velocity assessments are difficult to interpret and do not provide direct information on the flow of arterial blood into the capillary beds (CBF).

Blood flow in the supplying vessels of the brain can also be measured using phase-contrast MRI. With this technique, total CBF can be assessed by simultaneously measuring the flow in the internal carotid arteries and the basilar artery. This non-invasive MRI approach provides a measure of blood flow velocity in the feeding arteries that can be multiplied by the cross-sectional area of the vessels to determine blood flow to the whole brain [25]. 

NIRS is a non-invasive method that uses the absorption of near-infrared light introduced through the skull to reveal changes in CBF in surface layers of the cortex related to localized neural activity. This brain imaging technique is predicated on the intrinsic optical properties of oxygenated and deoxygenated hemoglobin and has been extensively used as a method to assess changes in CBF during task performance in relevant brain regions [26]. When assessed by NIRS, for example, an increase in blood flow in the prefrontal cortex during cognitive task-induced neural activity is observed as an increase in the total hemoglobin concentration and comparative decrease in deoxygenated hemoglobin. A key limitation, however, is that this qualitative CBF measurement depends on changes in both blood flow and oxygen metabolism, and only provides a measure of acute changes that have taken place during task performance.

## 4. Dietary Factors and Cerebral Blood Flow

### 4.1. Dietary Nitrate

Dietary nitrate, which is found in high concentrations in red beetroot, lettuce, and spinach, may improve CBF through beneficial effects on vascular endothelial function, which is an important mechanistic determinant of cerebral perfusion [27]. In the mouth, dietary nitrate (NO_3_^−^) can be reduced to nitrite (NO_2_^−^) by facultative bacteria from the dorsal surface of the tongue. Once in the blood, nitrite can be further converted into nitric oxide (NO) in the human vasculature, thereby improving endothelial function via an increased NO bioavailability [28]. Several human intervention studies have examined the acute effect of dietary nitrate intake on measures of CBF (see Table 1). Presley and colleagues [29] measured cerebral perfusion using ASL MRI after administering a high- versus low-nitrate diet for 24 h to a group of elderly humans (74.7 ± 6.9 years). The test diet included beetroot juice and provided 773 mg of nitrate compared to 5.5 mg for the low-nitrate diet. The authors demonstrated that the diet high in dietary nitrate did not significantly increase global CBF, while regional cerebral perfusion improved in frontal lobe white matter, especially between the dorsolateral prefrontal cortex and the anterior cingulate cortex, which are known to be involved in executive functioning. However, whether the observed increase in regional CBF in coincides with concurrent improvements in cognitive functioning remains to be elucidated. A single dose of 500 mL nitrate-rich beetroot juice (providing 750 mg dietary nitrate) acutely increased MCA mean blood flow velocity, measured non-invasively with transcranial Doppler ultrasonography during submaximal exercise in twelve healthy, normotensive young adult females [30]. More recently, Wightman et al. investigated the acute effects of 450 mL of beetroot juice (providing 342 mg of dietary nitrate) on prefrontal cortex CBF parameters in 40 apparently healthy adults. It was found that a single dose of beetroot juice modulated the NIRS-monitored CBF hemodynamic response during the performance of tasks, which activated the prefrontal cortex. In fact, an initial rise in prefrontal cortex perfusion at the start of the task period was followed by consistent reductions in cerebral perfusion during the least demanding task, while performance on one of the three cognitive tasks—requiring resources in terms of working memory, psychomotor speed and executive functioning—was improved [31].

### 4.2. Polyphenols

Polyphenols are predominantly found in fruits and vegetables, as well as red wine, tea and chocolate. These phytochemicals may exert beneficial effects on brain health due to their positive impact on endothelial function and other aspects of the vasculature through an increased NO bioavailability [32], which may translate into increased CBF [27]. Acute intake of trans-resveratrol, which is present in the skin of a range of foods including red grapes, raised CBF in healthy adults (Table 1). In a randomized, placebo-controlled, crossover study, trans-resveratrol (250 and 500 mg) administration resulted in dose-dependent increases in prefrontal cortex CBF during task performances which activated this brain region, as assessed with NIRS. The performance of cognitive tasks was not changed [33]. These results were in line with those of another clinical trial [34] on the acute effects of 250 mg trans-resveratrol co-supplemented with 20 mg of piperine, which increases the bioavailability of polyphenols. More recently, the effects of long-term trans-resveratrol supplementation on CBF were investigated in 60 adult subjects between the ages of 18 and 30 years. In that study, a single 500 mg dose of trans-resveratrol on the first day of the study increased the CBF response in the frontal cortex (NIRS) during tasks which activate this brain region. However, this effect was not observed with transcranial Doppler ultrasound parameters after 28 days of daily supplementation of 500 mg of trans-resveratrol [35]. In addition, no unambiguous evidence was provided to support that the intake of trans-resveratrol improved cognitive function. The effects of trans-resveratrol intake have also been investigated in populations at an increased risk of accelerated cognitive decline. In 36 older type 2 diabetic patients, acute consumption of 75 mg of trans-resveratrol significantly improved hypercapnia-induced mean blood flow velocity responses in major cerebral arteries by about 13% [36]. Also, Evans and colleagues reported the beneficial effects of long-term trans-resveratrol supplementation (75 mg twice daily for 14 weeks) in postmenopausal women aged 45–80 years [37]. In that study, increases of 17% were found in the MCA mean blood flow velocity response to cognitive stimuli and hypercapnia. In addition, performance of a cognitive task in the domain of verbal memory and in overall cognitive performance improved, which correlated with improvements in transcranial Doppler ultrasound parameters.

A smaller number of intervention trials has assessed the cerebral hemodynamic effects of other dietary polyphenols (see Table 1). In a double-blind, placebo-controlled, crossover study with 27 healthy adults, the acute effects of a single oral dose of epigallocatechin gallate—the most abundant polyphenol found in green tea—were investigated on CBF [38]. The administration of 135 mg of epigallocatechin gallate reduced cerebral perfusion (NIRS) in the frontal cortex during performance of cognitive tasks activating the frontal cortex, but no changes in cognitive performance were observed. More recently, a human crossover trial was conducted on the cerebrovascular effects of flavanol-rich cocoa [39]. Regional CBF was measured using ASL prior to and two hours following consumption of a high flavanol drink (494 mg) or placebo. In agreement with an RCT involving healthy young men (903 mg cocoa flavanols) [40] and a pilot trial of four healthy females consuming 516 mg of cocoa flavonoids [41], acute improvements in resting CBF were observed following the consumption of the high cocoa flavanol drink in eight men and ten women aged 50–65 years [39]. More specifically, higher resting CBF was observed in both the anterior cingulate cortex and the central opercular cortex of the left parietal lobe. Unfortunately, the effects on cognitive performance were not evaluated. In another study with elderly subjects, a single dose of cocoa (providing 900 mg of flavanols) increased hypercapnia-induced MCA blood flow velocity. The effects, however, were not evident after a daily supplementation of 900 mg of cocoa flavanols for one week [42]. Finally, Bowtell and colleagues investigated the effects of twelve weeks of blueberry concentrate supplementation on cerebral perfusion using ASL MRI in healthy elderly adults. The concentrate provided 387 mg of anthocyanins (major polyphenol pigments). They found improvements in gray matter CBF in the parietal and occipital lobes, as well as some evidence suggesting an improved working memory after blueberry versus placebo supplementation [43].

### 4.3. Dietary Fatty Acids

Haast and Kiliaan recently summarized the effects of the *n-3* long-chain polyunsaturated fatty acids (LC-PUFAs), eicosapentaenoic acid (EPA), and docosahexaenoic acid (DHA), which are predominantly found in fatty fish and fish oils, on indicators of brain health [44]. These dietary fatty acids can be incorporated in all lipid fractions. In fact, LC-PUFAs may have anti-inflammatory effects and increase the fluidity of cell membranes. They may also improve vascular endothelial function and arterial stiffness [45], which are both important mechanistic determinants of CBF [27]. Two human intervention trials investigating the longer-term effects of these fatty acids on CBF were discussed by Haast and Kiliaan [46,47]. One study indeed showed that regional cerebral perfusion in the prefrontal cortex improved during the performance of nine computerized cognitive tasks. In the study, 65 healthy adults received for 12 weeks a daily DHA-rich fish oil supplement of either 1000 or 2000 mg. The total daily dose of LC-PUFAs for the 1000 mg fish oil group was 450 mg DHA + 90 mg EPA, and for the 2000 mg fish oil group, these amounts were 900 mg DHA + 180 mg EPA [46]. Relative changes in the concentration of hemoglobin were assessed in the prefrontal cortex using NIRS. However, no beneficial effects on cognitive performance were found. Furthermore, PET experiments in humans that were injected intravenously with labeled DHA showed that the rate of DHA incorporation into brain lipids correlated with the regional CBF in that particular region [47].

Konagai and colleagues [48] provided further evidence of the beneficial modulation of cerebral hemodynamics in the prefrontal cortex during working memory task completion, when 45 elderly men were supplemented for 12 weeks with *n-3* LC-PUFAs from krill oil. However, these findings could not be replicated in a recent large RCT. In 86 healthy older adults who reported memory deficits, no effects of long-term (six months) supplementation of 2000 mg DHA-rich fish oil (896–946 mg DHA + 128–160 mg EPA) alone or in combination with other nutrients (i.e., Gingko biloba, phosphatidylserine, and vitamins B9 and B12) were observed on NIRS measures during task performance or on cognitive demand battery task outcomes [49]. These results, however, should be interpreted with caution. As discussed by the authors, the study was limited by the fact that the utilized methodology (NIRS) only provides a measure of acute changes that have taken place during each discrete recording session. This limitation should be taken into account in long-term (i.e., longer than 12 weeks) supplementation studies, because long-lasting changes in hemodynamic parameters between consecutive recording sessions—undetectable by NIRS—might be induced.

### 4.4. Caffeine

The most widely consumed psychoactive compound is caffeine, which is found in various drinks and foods, such as coffee, tea, soft drinks and chocolate. Caffeine is a well-known cerebral vasoconstrictor, which significantly reduces resting cerebral perfusion by antagonizing adenosine receptors in the human brain, especially A_1_ and A_2A_ subtypes that mediate vasodilation. By using PET methodology, Cameron et al. quantified the magnitude of the decrease in CBF in 1990 [50]. A single dose of 250 mg caffeine reduced resting CBF, with decreases ranging from 22% to 30%; this is in line with later studies using ASL and PET [51]. Recently, Turnbull and colleagues evaluated the literature with respect to the effects of acute caffeine intake (45 to 400 mg) on CBF in adult subjects [52]. Trials investigating intakes of ≥175 mg observed significant decreases in CBF in all study populations. Studies that administered lower doses only reported significant decreases in caffeine-naïve or low-caffeine consumers, but not in habitual consumers. Altogether, the authors of the recent review concluded that there is some evidence for a dose-response relationship between caffeine intake and CBF, with greater sensitivity in caffeine-naïve study subjects as compared to habitual caffeine consumers [52]. Interestingly, a global reduction by approximately 20% in gray matter CBF (measured with ASL perfusion MRI) was not only observed two hours after the intake of a single dose of 184 mg caffeine (equivalent to one strong espresso coffee), but also following the consumption of 2820 mg black tea solids containing 184 mg caffeine that is equivalent to about six cups of tea [53]. This suggests that flavonoids (~902 mg) in black tea did not affect the acute decrease in CBF following the intake of caffeine in healthy male subjects with a mean age of 24 years.

Even though a robust blood flow decrease in response to acute caffeine consumption was observed, neural activity was enhanced, as adenosine’s effects are antagonized [54]. Cognitive performance, however, was not changed. This might partly be explained by results from calibrated blood oxygenation level-dependent (BOLD) functional MRI experiments. It was shown that after caffeine intake, the cerebral metabolic rate of oxygen consumption remained stable in some [25,55], but not all studies [56], because the decrease in CBF was compensated for by an increase in oxygen extraction. More importantly, long-term effects are unclear. Vascular adenosine receptors may be upregulated during long-term caffeine use to preserve the CBF at a level that would have existed in a caffeine-naïve state. Another main issue are the effects of caffeine withdrawal. CBF may be abnormally high due to overnight withdrawal from caffeine or abnormally low due to recent caffeine ingestion. Due to its widespread use, caffeine is therefore a potential confounder in many cerebral perfusion studies, complicating the interpretation of the study results [57]. 

Interestingly, the observed effects of caffeine on CBF may explain its efficacy as a contrast booster in functional MRI studies. In fact, by acting as a cerebral vasoconstrictor, caffeine causes an increase in the concentration of deoxyhemoglobin, and thus decreases the BOLD baseline resting signal. During activation, the human vasculature responds from below-normal baseline levels with a normal increase in blood flow, resulting in an overall increase in the BOLD contrast. The benefit of an increased BOLD signal contrast can be used to improve, for example, image resolution, the acquisition scheme, or the task design of functional MRI experiments [58].

### 4.5. Alcohol

Neuroimaging studies clearly indicate that alcohol increases cerebral perfusion. In fact, alcohol may affect CBF directly via its vasodilatory effects on arterial vessels and may induce changes in brain metabolism that trigger indirect vasodilatory mechanisms. As reviewed by Bjork and Gilman [59], earlier studies using the dynamic SPECT technique with inhalation of Xenon-133 have indicated significant increases in mean gray matter CBF at low [60], moderate [61,62], and high alcohol doses [60]. Similar effects were found on global cortical CBF measured with [^99m^Tc]-HMPAO (hexamethylpropyleneamine oxime) SPECT following alcohol intoxication [63]. Volkow et al. performed blood flow measurements using ^15^O-labeleld water and PET. They demonstrated increased perfusion in the temporal and prefrontal cortex, while consumption of 1.0 g/kg of alcohol reduced blood flow in the cerebellum in a group of thirteen normal drinkers consuming 1 to 2 mixed drinks or 2 to 4 beers per week [64]. More recent intervention trials using MRI methods based on ASL perfusion contrast have further established that moderate alcohol doses of about 0.6 g/kg breath alcohol concentration (BrAC) increase CBF, although the between-subject and regional variabilities are large. In fact, Rickenbacher and colleagues indicated an increased perfusion in bilateral frontal regions after acute alcohol administration in men, but not in women [65], while Khalili-Mahani [66] reported a global perfusion increased in response to intravenously administered alcohol in only half of the subjects. In a trial involving a total of 88 healthy young adult social drinkers, cerebral perfusion increased after alcohol as compared to placebo in only frontal brain regions [67], whereas other RCTs also reported significant treatment differences in other brain regions [66,68]. The purpose of the study by Marxen et al. was to reduce potential sources of inter-subject variability [68]. In 48 young adults aged either 18 or 19 years, CBF was monitored continuously during (i) the rise of BrAC up to 0.6 g/kg and (ii) a steady BrAC of 0.6 g/kg for two hours. Interestingly, global perfusion increased after alcohol consumption by about 7% as Blood Alcohol Concentrations (BAC) approached 60 mg %, and CBF increases and changes in BrAC were tightly coupled in time. Except for the occipital lobe, significant increases in CBF were observed in most regions of the brain. These findings are in agreement with a more recent study that showed widespread and dose-dependent increases in cortical perfusion following intravenous alcohol administration [69]. In addition, measurements were performed at a higher BAC concentration of 80 mg %, which represents a clinically relevant threshold for heavy episodic alcohol exposure, as defined by the National Institute on alcohol abuse and alcoholism (NIAAA). It was reported that increases in cerebral perfusion were significantly more pronounced at 80 mg % versus 40 mg % for specific frontal brain regions, suggesting that these brain regions are particularly sensitive to the vasoactive effects of acute alcohol intake.

Evidence for the chronic effects of alcohol on CBF is limited, but observational studies found that long-term alcohol consumption was associated with reduced CBF in the sober state. Decreased perfusion levels were observed both globally and regionally in the frontal, temporal, parietal, and occipital cortices, as well as in the thalamus [70].

## 5. Exercise and Cerebral Blood Flow

As reviewed by Ogoh and Ainslie [71], cerebral perfusion increased during mild to moderate exercise intensities up to about 60% of maximal oxygen uptake. This is due not only to increases in cardiac output, but also to changes in neuronal activity and metabolism in regions associated with central command and skeletal muscle afferents. However, CBF during exercise is largely dependent on exercise intensity. In contrast to mild to moderate exercise, higher exercise intensities first increase cerebral perfusion, after which CBF reduces towards resting values, possibly due to hyperventilation-induced cerebral vasoconstriction. The effects of long-term exercise on CBF have hardly been studied [72]. In 307 healthy men aged 18 to 79 years, regular aerobic-endurance training was significantly associated with higher mean blood flow velocity in the MCA, measured with transcranial Doppler ultrasonography. In fact, a difference of approximately 17% in blood flow velocity was observed at rest between exercise-trained and sedentary men, independently of possible confounding variables [73]. The effects of a six-month aerobic exercise-based cardiac rehabilitation program on regional CBF were investigated in coronary artery disease patients [74]. In this trial using ASL, CBF increased in the bilateral anterior cingulate region, which is involved in cognitive processing. In addition, Chapman et al. [75] found increased CBF in the anterior cingulate region, and related increase of memory performance in 37 cognitively healthy sedentary adults after three months of supervised aerobic-based exercise training. This indicates that improvements in cerebrovascular function may be a mechanism underlying the beneficial effects of exercise on cognitive function.

## 6. Conclusions and Future Directions

In summary, CBF is a sensitive physiological marker of cerebrovascular function that can be quantified, for example, by the non-invasive MRI method ASL. ASL provides a highly repeatable quantitative measure of human brain perfusion [8,9]. Cerebral perfusion is positively associated with cognitive performance [17]. Furthermore, lifestyle factors, including dietary composition and physical exercise, can significantly increase CBF, thereby improving cognitive performance. A limited number of studies have observed the beneficial effects of acute intakes of dietary nitrate and polyphenols on CBF. However, evidence for a significant relationship between these effects and improvements in cognitive functioning is limited, possibly because acute perfusion changes are not related to improvements in cognitive function in healthy young adults, who were involved in most of these intervention studies. Furthermore, long-term trans-resveratrol supplementation enhanced CBF in populations at increased risk of accelerated cognitive decline. A long-term supplementation of *n-3* LC-PUFAs may also increase CBF, but the related effects on cognitive performance have not yet been found in these supplementation studies. Consistent evidence exists for the acute effects of caffeine and alcohol. Decreases in CBF are induced by commonly consumed amounts of caffeine, while alcohol significantly increases cerebral perfusion in a dose-dependent way. Long-term effects of caffeine and alcohol, however, are not clear. Finally, long-term exercise training may be a promising future approach to improve CBF, as it is thought that increases in perfusion contribute to the beneficial effects on cognitive functioning following increased physical activity levels.

**Figure 3:**
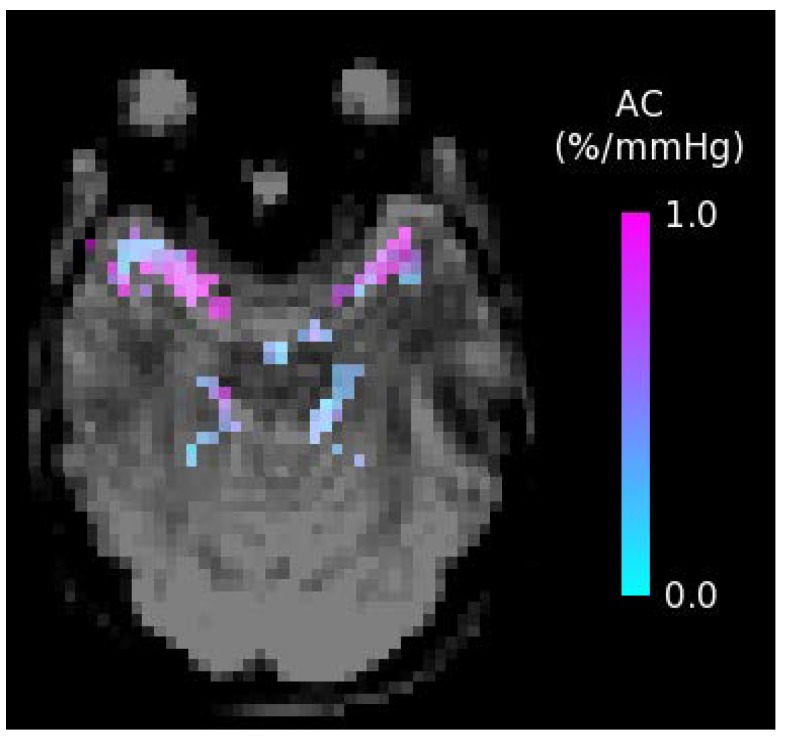
Arterial Compliance (AC) map showing the percentage change in arterial blood volume per millimeter of mercury (%/mmHg); obtained using short inversion time arterial spin labeling.

Future well-designed RCTs are still warranted to further investigate the potential of diet and exercise on CBF. Of particular use would be longer-term intervention studies focusing on middle-aged and elderly adults at increased vascular risk who are also known to be at increased risk of cognitive impairment and dementia. Furthermore, the specific effects of lifestyle changes on other physiological measures of cerebrovascular function are largely unknown. These effects are also of major interest, since other aspects of an impaired brain vascular function may also consist of key pathological events that precede the development of impaired cognitive function. Understanding the potential of lifestyle factors to affect these outcome measurements may further help to determine effective strategies to mitigate the adverse effects of human aging on cognitive performance. Future research should thus broaden its focus, considering emerging MRI techniques for the non-invasive assessment of additional brain vascular measures. For example, the dietary and physical exercise effects on the compliance (arterial stiffness) of the major cerebral arteries measured non-invasively with short inversion time ASL [76] and the cerebrovascular reactivity to hypercapnia (endothelial function) [77] are of great interest. An example arterial compliance map is shown in Figure 3, with compliance in units of percentage change in arterial blood volume per millimeter of mercury (%/mmHg). From these maps, measures of average arterial compliance can be obtained in major cerebral arteries, including the left middle, right middle, left posterior, right posterior, and anterior cerebral arteries, in a region just superior to the circle of Willis that supplies blood to the brain.

## Figures and Tables

**Table 1:** Effects of dietary nitrate and polyphenols on cerebral blood flow.

Author, Year	Study Design	Treatment	Dose	Duration	Study Population (*n*)	Effect on CBF ^1^	Method	Effect on Cognition
**Dietary Nitrate**								
Presley, 2011 [29]	Randomized, controlled crossover trial	High-nitrate diet (2000 kcal/day)	773 mg/day	24 h	Older adults (14)	No effect on global CBF, but increased regional CBF in frontal lobe white matter	ASL MRI	(not evaluated)
Bond, 2013 [30]	Randomized, controlled crossover trial	Nitrate-rich beetroot juice (500 mL)	750 mg	Acute	Healthy young women (12)	Increased MCA blood flow velocity during submaximal aerobic-based exercise	Doppler sonography	(not evaluated)
Wightman, 2015 [31]	Randomized, double-blind, placebo-controlled parallel trial	Nitrate-rich beetroot juice (450 mL)	342 mg	Acute	Healthy young adults (40)	Modulated CBF in the prefrontal cortex during cognitive task performance	NIRS	Improved performance on one cognitive task
**Polyphenols**								
Kennedy, 2010 [33]	Randomized, double-blind, placebo-controlled crossover trial	trans-Resveratrol	250 and 500 mg	Acute	Healthy young adults (22)	Increased CBF (dose-dependent) in the prefrontal cortex during cognitive task performance	NIRS	No effect on cognitive performance
Wightman, 2014 [34]	Randomized, double-blind, placebo-controlled crossover trial	trans-Resveratrol + piperine (20 mg)	250 mg	Acute	Healthy young adults (23)	Increased CBF in the prefrontal cortex during cognitive task performance	NIRS	No effect on cognitive performance
Wightman, 2015 [35]	Randomized, double-blind, placebo-controlled parallel trial	trans-Resveratrol	500 mg/day	28 days	Healthy young adults (60)	Acutely increased CBF in the prefrontal cortex during cognitive task performance, but no chronic effects	NIRS	No effect on cognitive performance
Wong, 2016 [36]	Randomized, double-blind, placebo-controlled crossover trial	trans-Resveratrol	75, 150 and 300 mg	Acute	Older type 2 diabetics (36)	Increased hypercapnia-induced MCA blood flow velocity response	Doppler sonography	(not evaluated)
Evans, 2017 [37]	Randomized, double-blind, placebo-controlled parallel trial	trans-Resveratrol	150 mg/day	14 weeks	Postmenopausal women (80)	Increased cognitive task/hypercapnia-induced MCA blood flow velocity response	Doppler sonography	Improved overall cognitive performance
Wightman, 2012 [38]	Randomized, double-blind, placebo-controlled crossover trial	Epigallocatechin gallate	135 mg	Acute	Healthy young adults (27)	Reduced CBF in the prefrontal cortex during cognitive task performance	NIRS	No effect on cognitive performance
Lamport, 2015 [39]	Randomized, double-blind, placebo-controlled crossover trial	Cocoa flavanols	494 mg	Acute	Older adults (18)	Increased regional CBF in the anterior cingulate cortex and central opercular cortex	ASL MRI	(not evaluated)
Decroix, 2016 [40]	Randomized, double-blind, placebo-controlled crossover trial	Cocoa flavanols	903 mg	Acute	Healthy young men (12)	Increased CBF in the prefrontal cortex during cognitive task performance	NIRS	No effect on cognitive performance
Sorond, 2008 [42]	Randomized, double-blind, placebo-controlled parallel trial	Cocoa flavanols	900 mg/day	1 week	Older adults (21)	No effect on the hypercapnia-induced MCA blood flow velocity response	Doppler sonography	(not evaluated)
Bowtell, 2017 [43]	Randomized, double-blind, placebo-controlled parallel trial	Anthocyanin-rich blueberry concentrate	387/day	12 weeks	Older adults (26)	Increased regional CBF in parietal and occipital lobe gray matter	ASL MRI	Improved performance on one cognitive task

^1^ CBF: cerebral blood flow; ASL: arterial spin labeling; MRI: magnetic resonance imaging: MCA: middle cerebral artery; NIRS: near-infrared spectroscopy.
